# Annotations

**Published:** 1908-09-19

**Authors:** 


### Annotations.

### Educational Science.

Professor L. C. Miall, F.R.S., at the meeting of the British Association in Dublin, showed that hitherto, in order to prepare pupils speedily for examination, all the hard work has to be done by the teacher, who is supposed to prepare peptonised food for their weak intellects. Such a procedure can only lead to a want of education and an entire absence of self-reliance. In particular it seems to him that the preliminary scientific studies of the medical man are inadequate both in scope and depth. These studies are not sufficiently connected with the study of medicine to be of great value either in themselves or as an educatory medium. Education should, as it were, be conducted round a given point, in so far as it concerns a fundamental principle of science. The student and the teacher should bear in mind the object of their studies, and abandon as much as possible the routine training for pure routine's sake. Luckily, too, there are signs that educational authorities are beginning to realise the necessity for using the peculiar bent and tendency of the pupil. It will soon be considered absurd to make a boy a classic who has early exhibited great love for animals and the freer life of Nature. General opinion in the matter seems to give assent to the conclusion drawn by Herbert Spencer, that from whatever point of view one handles the question, science is the best means and object of education. But it must be remembered that, though a knowledge of scientific fact is not desirable as an ultimate end, it is very necessary as a means to an end. In any branch of science there will always be a certain amount of detailed fact which must be mastered before the student can hope to acquire a complete grasp of his subject. Referring again to medical education, it scarcely seems that enough emphasis is placed upon the need for a training conducive to self-reliance. The speaker hopes that it will be possible to give a student a thorough education along the principles laid down by Pasteur as fundamental in surgery, medicine, and hygiene. There is a most cheering optimism about this utterance on so vexed a question. It is a pleasing reaction against the slipshod indifference which seems to pervade educational minds.

### Newspaper Medicine.

It is fast becoming the duty of any medical worker who wishes to keep abreast of his subject to search carefully the lay press. Certainly no cancer researcher should miss the account in *The Scotsman* of September 10, of the annual meeting of the Glasgow Cancer and Skin Institution. It is true that their informant will be an Edinburgh lawyer, but what of this if, the ætiology of cancer being now known to be "sparks from locomotives," or "something that injured the nerves at a spot," there need be "no further investigation as to that, and the Imperial Cancer Research Committee ought to own the existence of cancer as due to such causes, and direct their energies to the necessary remedies for its elimination." But surely this latter task is also superfluous, since we learn that of sixty-five new cancer patients admitted during the year, all of whom had been undoubtedly suffering from true cancer, in most cases of a recurrent form, fifty-five were "discharged well." There is a touch of national caution in the use of this trifling circumlocution which is unworthy of the tone of the rest of the report. But in what scientific archives, one wonders, are these results to be preserved? And where can one get particulars of the "medical treatment" used? For there is no hope whatever of surgical operations ever removing cancer—a Solicitor of the Supreme Court has said so. He even thinks they do harm, for seven of the cases who died were even before admission "almost hopeless, as the result of repeated operations and the destruction of their constitutions." However, even this difficulty should not baffle for long those who have already done so much. Perhaps next year we may hear of a means of obviating the ill-effects of the knife nearly as good as the "sympathetic powder" of Kenelm Digby, which precious substance—something like a remedy!—would heal a wound when applied to the weapon that had caused it. People who propound their conclusions in this sort of way must not be surprised at an unpleasant reception.

### The American Hospital Conference.

The tenth annual Conference of the American Hospital Association for the Promotion of Economy and Efficiency in Hospital Management will be held at the King Edward Hotel, Toronto, Canada, on September 29 and 30 and October 1 and 2 of this year. The presidential address will be delivered on September 29 by Dr. S. S. Goldwater, of the Mount Sinai Hospital, New York; and the Provincial Secretary of Ontario, the Hon. W. J. Hanna will deliver an address of welcome. Many important papers will be read during the Conference, among which may be mentioned: "Problems in the Management of Small Hospitals," by Theodore R. MacClure, M.D.; "Some Scientific Aspects of Hospital Management," by John A. Hornsby, M.D., of Chicago; "Co-operation in Dispensary Work as Exemplified by the Association of Tuberculosis Clinics of New York," by James Alex. Miller, M.D., of New York; "The Development of the Work and the Restriction of the Abuse of the Out-patient Department," by (*a*) John M. Peters, M.D., (*b*) Frederick A. Washburn, M.D., (*c*) Thomas Howell, M.D., and (*d*) W. O. Mann, M.D.; "The Planning and Construction of Hospitals for Smaller Cities and Towns" (illustrated), by Meyer J. Sturm, architect, of Chicago; "Infectious Diseases in General Hospitals: Their Proper Control from the Standpoint of Sanitary Science," by Robert J. Wilson, M.D., Superintendent of Hospitals, Department of Health, City of New York; "The Care of the Minor Contagious Diseases," by Charles Sheard, M.D., Medical Health Officer, Toronto. In addition to these papers and discussions there will be receptions and visits to hospitals. The Local Committee of Arrangement have secured special tariffs at hotels for the benefit of members, and from every point of view the Congress promises to be a great success.

